# “How Comes It, Rocinante, You’re so Lean?” “I’m Underfed, with Overwork I’m Worn”

**DOI:** 10.3201/eid1409.090000

**Published:** 2008-09

**Authors:** Polyxeni Potter

**Affiliations:** Centers for Disease Control and Prevention, Atlanta, Georgia, USA

**Keywords:** Art science connection, emerging infectious diseases, art and medicine, homeless, poverty, infectious diseases, Bartolomé Esteban Murillo, Spanish baroque art, The Young Beggar, about the cover

**Figure:**
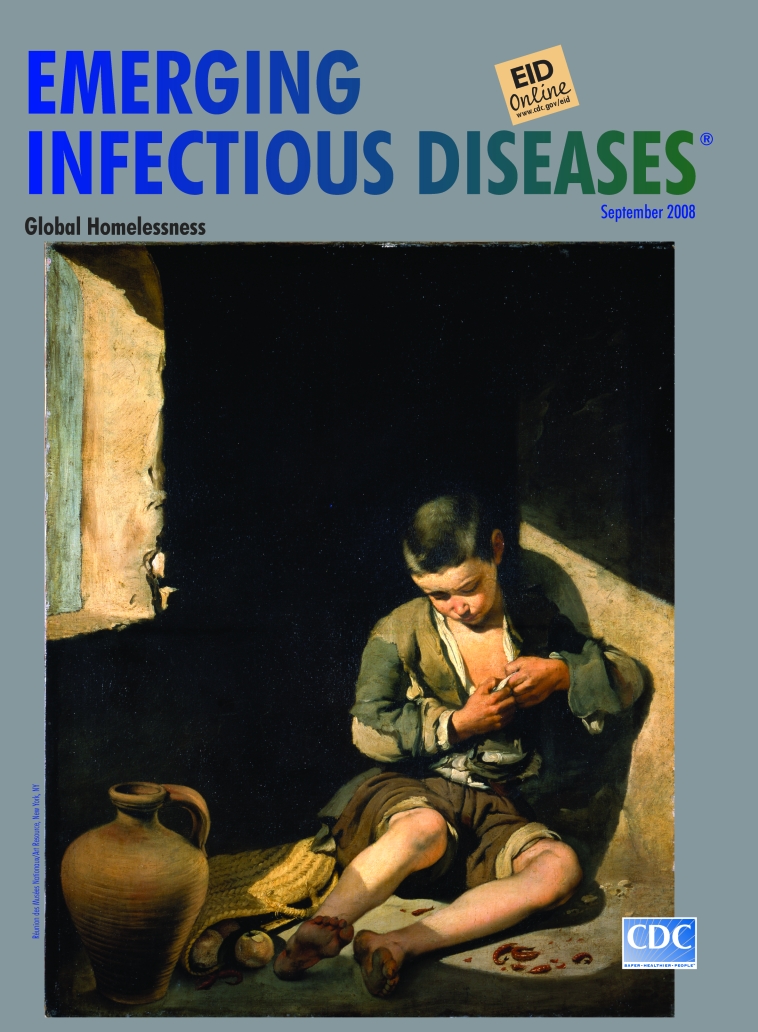
**Bartolomé Esteban Murillo (1617–1682) The Young Beggar (c. 1650)** Oil on canvas (1.34 m × 1 m) Photo: C. Jean. Louvre, Paris, France. Réunion des Musées Nationaux/Art Resource, New York, NY

―Cervantes

“There are only two families in the world, the Haves and the Haven’ts,” wrote Miguel de Cervantes (1547–1616) at a time he viewed as “no golden age” in his native Spain (*1*). A brilliant satirist, Cervantes ridiculed the socially divisive mores of a gilded imaginary past held onto for too long and seeming all the more incongruous amidst the poverty and oppression of his own life.

Spain in the 17th century, its empire collapsed and population ravaged by three plague epidemics, was embroiled in conflict abroad and royal mismanagement at home. Its misadventures, chronicled in the plays of Lope de Vega and Calderón, also fueled the genius of Diego Velázquez, Francisco de Zurbarán, Jusepe de Ribera, and Bartolomé Esteban Murillo. They, too, advanced the cultural front by constructing from the rabble a Spanish school of painting for the ages.

Spanish baroque, as the school came to be known, expanded on similar art movements in the Netherlands and elsewhere in Europe. Immortalizing royals and street peddlers alike, it left a vivid record of the times. Among the greats of this period, Murillo, a native of Seville known as much for his good character as his artistic talent, painted the indigent and populated lofty religious scenes with ordinary human faces.

Orphaned in childhood, Murillo was raised by relatives (*2*). He apprenticed under Juan del Castillo, a leading painter of the 1630s and 1640s who, along with Alonso Cano, influenced his early work. He left this apprenticeship to go into business for himself, creating popular pictures for quick sale. As a street artist, he saw the poorest in the city, so his early paintings were sympathetic portraits of the ragged boys and flower girls of Seville. His break came with a commission to paint 11 works for a local Franciscan monastery. These paintings brought him fame, and soon he was affluent enough to marry well.

He traveled to Madrid, where he likely became familiar with the work of Velázquez, fellow Sevillian and painter to the king, and the work of Rubens, Titian, Veronese, and Tintoretto in the royal galleries. He returned to his hometown to found the Seville Academy of Art, paint major works, and enjoy great popularity, even outside Spain (*3*). His mature paintings were mostly religious. Some were genre scenes with children, a novel theme. A few were fine portraits. All attained such lightness and refinement they were labeled “vaporous.”

Even now, a “Murillo” stands for a good painting in Spain. The artist made an impression, although his fortunes faltered over time. His works sold so well that the king limited their export. But they were copied too often. Poor imitations in a flooded market damaged his reputation, especially since he never signed or dated his paintings.

One legend has it that Murillo died poor; another, that he gave his wealth to charitable causes. His last work, the Espousal of St. Catherine, commissioned for a Capuchin monastery in Cadiz, was not completed. The painter fell from the scaffold and was severely injured. He was taken back to Seville, where his death was attributed to these injuries.

The Young Beggar, on this month’s cover, exemplifies Murillo’s technique. It showcases the masterful brushwork, use of chiaroscuro, meticulous attention to naturalism, and gentleness toward his subjects that attracted the attention of later artists, among them 18th- and 19th-century’s Sir Joshua Reynolds, John Constable, and Édouard Manet.

“Masterless children,” a common sight in Seville, were pitied and loathed by local society, which gave them alms with one hand and dismissed them with the other. Survival and socialization on the street suited them for servile tasks, which inevitably led to adult criminal opportunities, gambling, and prostitution (*4*). In 1593, the city was “full of small boys who wander about lost and begging and dying of hunger and sleep in doorways and on stone benches by the walls, poorly dressed, almost nude, and exposed to many dangers…and others have died of freezing by dawn” (*5*).

An unpublished parish-by-parish survey of the “honorable poor” of Seville in 1667 found that untended children aggregated in courtyards and abandoned buildings. Exposed to weather and pests, unwashed and malnourished, they were susceptible to infectious diseases, from ringworm to bubonic plague. When, against all odds, it snowed twice in 1624 and 1626, they were decimated. One study in the parish of San Bernardo showed that 27% of burials in 1617 to 1653 were of children.

Seville’s population declined in the 17th century. A 1679 silk merchants’ quarter survey showed that 40% of the buildings were vacant. Two serious plague epidemics and extensive emigration to the New World reduced the number of children. Charity, documented in literary works and prominent in the paintings of Murillo, was thought as noble and pious, but the ugliness of want (festered wounds, parasites, filth) so vivid in these paintings exposed the dark side. The poor had to register for licensed begging to be protected from impostors that would take “alms from the truly poor who cannot work” (*5*). City authorities used charitable funds to create “hospitals” for vagrants to be put away from the public eye.

A slant of light reminiscent of Caravaggio (1571–1610) illuminates Murillo’s Young Beggar, allowing a glimpse of his life. Weary and resigned, the boy leans against the wall, his belongings strewn along with the remnants of a meal, scraps of fish and rotting fruit. He is delousing himself. The mild demeanor of the child is punctuated by the furrowed brow and soiled feet. Like Rocinante, he is underfed, overworked, and riddled with pests.

“The Haves and the Haven’ts” are still at it. The Haves contributing to charity, the Haven’ts flooding the streets, some 100 million of them around the globe: migrant workers in People’s Republic of China and elsewhere, homeless in Los Angeles, Bristol, or Marseille. Authorities still sort the truly and deserving poor from impostors, while the ugliness of want (lice, tuberculosis, flu, HIV/AIDS, hepatitis, diphtheria) is not shrinking from public view (*6*,*7*).

The pathos in Murillo’s Young Beggar lies in the child’s complete abandon and his charm, unblemished by the circumstances. Well addressed in literature and art, the plight of the poor, its abatement and elimination, is a main concern of public health. Armed with time-honored public health practice: health education, prescription and device distribution, tuberculosis screening and treatment, improved hygiene, ivermectin use against scabies and body louse infestation, and systematic immunization, the public health worker can now join other dreamers championed by Cervantes in declaring, “ ‘My armour is my only wear, / My only rest the fray’ ” (*1*).
